# Targeting subtype in ALL

**DOI:** 10.1097/HS9.0000000000000271

**Published:** 2019-06-30

**Authors:** Arend von Stackelberg

**Affiliations:** Charité, Berlin, Germany

**Learning goals**

- Genetic alterations are important prognostic factors in ALL.
- Affected intracellular pathways may serve as therapeutic targets.
- Prospective trials to prove the efficacy and safety of targeted therapies are needed.

## Introduction

Acute lymphoblastic leukemia (ALL) is the most frequent malignant disease in children. With conventional chemotherapy, prognosis is favorable, however at the cost of acute and long-term side effects. Relapsed and refractory ALL in contrast is associated with poor prognosis. Therefore, in general, there is a medical need for targeted potentially less toxic therapy to replace toxic chemotherapy and overcome resistance.

In B-cell progenitor (BCP) ALL favorable genetic alterations are hyperdiploidy or translocation t(12;21)/ETV6-RUNX1, whereas t(9;22)/BCR-ABL1, KMT2A (MLL) rearrangements, chromosome 21 amplification (iAMP21), t(17/19)/TCF3-HLF and hypodiploidy are associated with poor prognosis. Numerous additional coexisting genetic lesions have been detected involving B-cell development, proliferation and differentiation pathways that may serve as prognostic markers or therapeutic targets.

In T-ALL, early T-immunophenotype (ETP) shows an immature genetic profile with stem-cell- and myeloid characteristics. Alterated pathways include NOTCH signaling, cell cycle regulation, PI3K, JAK/STAT, which may serve as therapeutic targets.

Targeted treatment may however be impaired by heteroclonality of the ALL. Prospective controlled trials to prove safety and efficacy of these approaches are largely lacking.

